# Paenibacillus odorifer, the Predominant *Paenibacillus* Species Isolated from Milk in the United States, Demonstrates Genetic and Phenotypic Conservation of Psychrotolerance but Clade-Associated Differences in Nitrogen Metabolic Pathways

**DOI:** 10.1128/mSphere.00739-19

**Published:** 2020-01-22

**Authors:** Sarah M. Beno, Rachel A. Cheng, Renato H. Orsi, Diana R. Duncan, Xiaodong Guo, Jasna Kovac, Laura M. Carroll, Nicole H. Martin, Martin Wiedmann

**Affiliations:** a Department of Food Science, Cornell University, Ithaca, New York, USA; b Department of Agrotechnology and Food Science, Wageningen University, Wageningen, Netherlands; c Department of Food Science, Pennsylvania State University, State College, Pennsylvania, USA; University of California, Davis

**Keywords:** *Paenibacillus odorifer*, whole-genome sequencing, psychrotolerance, nitrogen fixation, nitrogen metabolism

## Abstract

*Paenibacillus* is a spore-forming bacterial genus that is frequently isolated from fluid milk and is proposed to play a role in spoilage. To characterize the genetic and phenotypic diversity of *Paenibacillus* spp., we first used *rpoB* allelic typing data for a preexisting collection of 1,228 *Paenibacillus* species isolates collected from raw and processed milk, milk products, and dairy environmental sources. Whole-genome sequencing (WGS) and average nucleotide identity by BLAST (ANIb) analyses performed for a subset of 58 isolates representing unique and overrepresented *rpoB* allelic types in the collection revealed that these isolates represent 21 different *Paenibacillus* spp., with P. odorifer being the predominant species. Further genomic characterization of *P. odorifer* isolates identified two distinct phylogenetic clades, clades A and B, which showed significant overrepresentation of 172 and 164 ortholog clusters and 94 and 52 gene ontology (GO) terms, respectively. While nitrogen fixation genes were found in both clades, multiple genes associated with nitrate and nitrite reduction were overrepresented in clade A isolates; additional phenotypic testing demonstrated that nitrate reduction is specific to isolates in clade A. Hidden Markov models detected 9 to 10 different classes of cold shock-associated genetic elements in all *P. odorifer* isolates. Phenotypic testing revealed that all isolates tested here can grow in skim milk broth at 6°C, suggesting that psychrotolerance is conserved in *P. odorifer*. Overall, our data suggest that *Paenibacillus* spp. isolated from milk in the United States represent broad genetic diversity, which may provide challenges for targeted-control strategies aimed at reducing fluid milk spoilage.

**IMPORTANCE** Although *Paenibacillus* species isolates are frequently isolated from pasteurized fluid milk, the link between the genetic diversity and phenotypic characteristics of these isolates was not well understood, especially as some *Bacillales* isolated from milk are unable to grow at refrigeration temperatures. Our data demonstrate that *Paenibacillus* spp. isolated from fluid milk represent tremendous interspecies diversity, with P. odorifer being the predominant *Paenibacillus* sp. isolated. Furthermore, genetic and phenotypic data support that *P. odorifer* is well suited to transition from a soil-dwelling environment, where nitrogen fixation (and other nitrate/nitrite reduction pathways present only in clade A) may facilitate growth, to fluid milk, where its multiple cold shock-associated adaptations enable it to grow at refrigeration temperatures throughout the storage of milk. Therefore, efforts to reduce bacterial contamination of milk will require a systematic approach to reduce *P. odorifer* contamination of raw milk.

## Abstract (precis)

Although *Paenibacillus* species isolates are frequently isolated from pasteurized fluid milk, the link between the genetic diversity and phenotypic characteristics of these isolates was not well understood, especially as some *Bacillales* isolated from milk are unable to grow at refrigeration temperatures. Our data demonstrate that *Paenibacillus* spp. isolated from fluid milk represent tremendous interspecies diversity, with P. odorifer being the predominant *Paenibacillus* sp. isolated. Furthermore, genetic and phenotypic data support that *P. odorifer* is well suited to transition from a soil-dwelling environment, where nitrogen fixation (and other nitrate/nitrite reduction pathways present only in clade A) may facilitate growth, to fluid milk, where its multiple cold shock-associated adaptations enable it to grow at refrigeration temperatures throughout the storage of milk. Therefore, efforts to reduce bacterial contamination of milk will require a systematic approach to reduce *P. odorifer* contamination of raw milk.

## INTRODUCTION

*Paenibacillus* represents a highly diverse bacterial genus, encompassing at least 240 validated species (1). While some *Paenibacillus* spp. have been isolated from human clinical infections, suggesting that some strains may be opportunistic pathogens (2), the vast majority of *Paenibacillus* spp. are isolated from the environment (3) and have been shown to play an important symbiotic role in plant growth through their capacity to fix atmospheric nitrogen to ammonia (4, 5). Thus, the potential use of N_2_-fixing *Paenibacillus* spp. as biofertilizers to enhance the growth of agricultural crops through increased nitrogen uptake (NH_4_^+^ and NO_3_^−^) and metabolism has been proposed (6–9).

*Paenibacillus* spp. are commonly isolated from pasteurized refrigerated fluid milk, making this genus a primary concern for milk processors, as milk’s near-neutral pH and storage at low temperatures for extended periods of time allow *Paenibacillus* spp. to grow to high levels. While sensory analyses of milk that has been artificially contaminated with *Paenibacillus* spp. have not been formally conducted to confirm *Paenibacillus*’ role in spoilage, because some *Paenibacillus* spp. can grow to high levels and cause proteolysis and lipolysis at refrigeration temperatures (3, 10–12), members of this genus are proposed to be key contributors to fluid milk spoilage. This is particularly poignant as USDA estimates suggest that 17 billion pounds of fluid milk (32%) are discarded at the retail/consumer levels each year in the United States (13). Furthermore, as *Paenibacillus* spp. are capable of forming endospores that survive high-temperature short-time pasteurization (e.g., 72°C for at least 15 s), conventional methods aimed at ensuring the safety of fluid milk may fail to control *Paenibacillus* species contamination if spores, which can survive these heat treatments and then germinate in refrigerated milk, are present.

The ability to grow at low temperatures is conserved for a number of different bacterial genera and includes what is known as the cold shock response (14), which is typically defined for mesophilic bacteria as the transition from a favorable temperature (e.g., 30°C to 37°C) to a lower, less favorable temperature (usually <15°C). At low temperatures, transcription and translation are impeded due to secondary structures that form in the RNA. To overcome this, bacteria produce nucleic acid chaperones, such as cold shock proteins (CSPs) that contain the cold shock domain (CSD), to maintain RNA in an unstructured state to allow translation to occur (15). Other cold shock responses include (i) the expression of fatty acid (FA) hydroxylases, which effectively change the fatty acid profile of the cell membrane to maintain fluidity at low temperatures (16); (ii) the production of chaperones that ensure proper folding of proteins at low temperatures (14, 17); and (iii) the production of DEAD box RNA helicases to unfold the secondary structures of double-stranded RNA, which may occur at low temperatures (15).

The majority of characterizations of bacterial cold shock responses have been conducted using Escherichia coli and Bacillus subtilis, and little is known about these responses in *Paenibacillus* species isolates despite their documented importance as psychrotolerant bacteria that are often associated with spoilage (18). Dsouza et al. reported the presence of *cspB* and *cspC* (encoding the cold shock proteins CspB and CspC, respectively) in three Paenibacillus darwinianus isolates from Antarctica as well as in nine temperate *Paenibacillus* species isolates (19). Furthermore, Moreno Switt et al. detected a DEAD box helicase present in a Paenibacillus amylolyticus isolate that was able to grow in skim milk broth (SMB) at 6°C (20). Still, our understanding of the types of cold shock responses encoded in other agriculturally relevant *Paenibacillus* spp. remains limited.

To better understand the genetic and phenotypic diversity of *Paenibacillus* spp. commonly isolated from fluid milk, we (i) used *rpoB* allelic typing to assess the diversity of 1,228 *Paenibacillus* species isolates collected from dairy products (predominantly fluid milk) and dairy environments, (ii) characterized a subset of these *Paenibacillus* spp. by whole-genome sequencing (WGS), and (iii) performed phenotypic analyses to associate genotypes with growth and metabolic characteristics.

## RESULTS

### *P. odorifer* is the predominant *Paenibacillus* sp. among a collection of 1,228 isolates collected from milk and dairy-associated environments.

**FIG 1:**
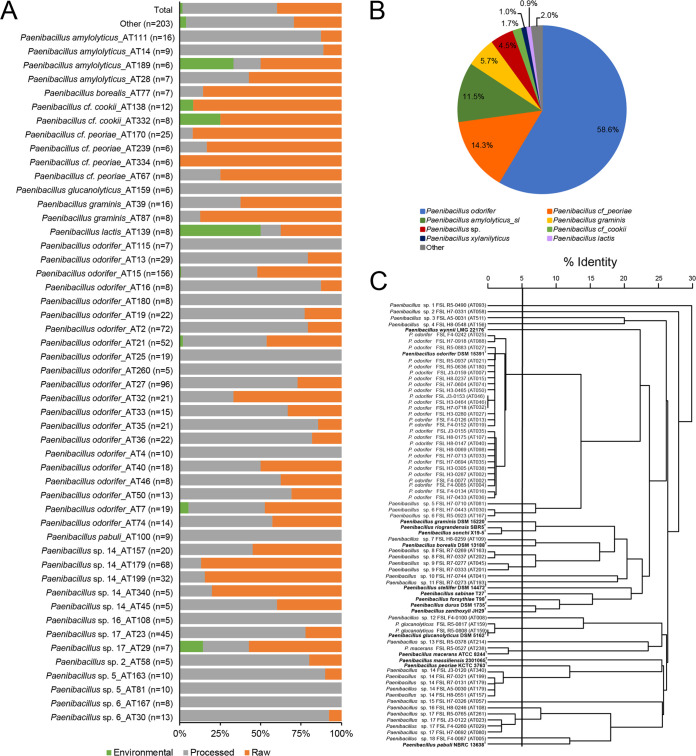
*Paenibacillus* spp. isolated from fluid milk and dairy-associated environmental sources in the United States represent tremendous genetic diversity. (A) Bar chart displaying the sources of different *rpoB* allelic types of *Paenibacillus* species isolates represented five or more times among a collection of 1,228 *Paenibacillus* isolates. Colors correspond to the isolation source, and the length of each colored section of the bar reflects the percentage of isolates from a given source. (B) Distribution of *Paenibacillus* spp. identified using a 632-nt internal sequence of *rpoB*. Isolates in the “other” category represent a total of 24 isolates (representing the species *P. glucanolyticus*, *P. lautus*, *P. pabuli*, *P. macerans*, *P. castanae*, *P. jilunlii*, *P. cineris*, and *P. illinoisensis*). Isolates in the “*Paenibacillus* sp.” category represent isolates with *rpoB* alleles that could not be differentiated to the species level. (C) ANIb analysis of a subset of 58 *Paenibacillus* species isolates. Isolates shown in boldface type represent type strains. The vertical line represents the 95% cutoff for species identification.

*Paenibacillus* spp. have previously been associated with spoilage of fluid milk and other dairy products (3, 12, 21). To assess the diversity of *Paenibacillus* spp. isolated throughout the dairy production chain, we characterized our collection, including 1,228 *Paenibacillus* species isolates that were identified based on *rpoB* allelic typing data from previously reported (10, 22–26) and ongoing (our unpublished data) studies. Overall, the 1,228 *Paenibacillus* species isolates comprised 177 unique *rpoB* allelic types (ATs). While most ATs were isolated from both raw and processed milk, some ATs were isolated throughout the dairy production chain, for example, from animal feed, cow teat swabs, raw milk, and pasteurized milk (Fig. 1A). The majority (58.6%) of the *Paenibacillus* species isolates in the collection (136 *rpoB* ATs) were obtained from processed fluid milk and milk products. Raw milk was the second most common source, with 39.7% of *Paenibacillus* isolates (82 *rpoB* ATs) in the collection being obtained from raw milk. The remaining 1.7% of isolates were obtained from environmental sources and represented 16 *rpoB* ATs. Based on *rpoB* sequence data, the majority of the *Paenibacillus* species isolates were classified into the species P. odorifer (58.6%), P. peoriae (14.3%), or *P. amylolyticus* (11.5%) (Fig. 1B). The remaining ∼15% of isolates represented isolates of the species P. graminis, P. cookii, P. xylanilyticus, P. lactis, P. glucanolyticus, P. lautus, P. pabuli, P. macerans, P. castanae, P. jilunlii, P. cineris, and P. illinoisensis and 24 isolates that could not be identified to the species level using *rpoB* allelic typing (Fig. 1B). Together, these results indicate that fluid milk harbors a diverse collection of *Paenibacillus* species isolates.

### ANIb analyses identified a number of potentially novel *Paenibacillus* spp.

To provide further resolution for *rpoB* ATs that were common in our collection and to also provide species identification for isolates that could not be assigned a species designation based on *rpoB* allelic typing, we performed WGS for 58 *Paenibacillus* isolates (Table 1). Initial average nucleotide identity by BLAST (ANIb) analyses of the genome sequences of the 58 *Paenibacillus* isolates characterized here along with 16 sequences representing relevant type strains (Fig. 1C) were performed to verify genus and species assignments. Using a 95% cutoff for ANIb classification into the same species (27), the ANIb analyses indicated that the subset of 58 dairy *Paenibacillus* isolates represented 21 different *Paenibacillus* spp., with the majority of isolates (*n* = 27) representing *P. odorifer* (Fig. 1C); *P. glucanolyticus* (*n* = 2 isolates) and *P. macerans* (*n* = 1) were also identified (Fig. 1C). The remaining 28 isolates represented 18 additional *Paenibacillus* spp. that could not be reliably classified to the species level, as they did not show similarities of ≥95% with any of the type strain genomes included in our analyses. Interestingly, two *Paenibacillus* type strains used in the ANIb analyses (P. riograndensis SBR5^T^ and P. sonchi X19-5^T^) show an ANIb similarity score of 98% and hence do not meet the species cutoff of <95% similarity, suggesting that these strains belong to the same species. Together, these analyses suggest that milk and dairy environments include a number of uncharacterized and unnamed *Paenibacillus* spp.

**TABLE 1:** Detailed information for the 58 *Paenibacillus* isolates characterized by whole-genome sequencing

Strain	Genus and species	*rpoB* AT	Source	Assembly size (Mb)	Avg nucleotide coverage	SRA accession no.^*a*^	WGS NCBI accession no.
FSL A5-0030	*Paenibacillus* sp.	179	Pasteurized milk	6	94	SRR4434617	MRTC00000000
FSL A5-0031	*Paenibacillus* sp.	511	Pasteurized milk	7.8	81	SRR4434616	MRTD00000000
FSL F4-0077	Paenibacillus odorifer	2	Pasteurized milk	6.9	102	SRR4434615	MPVO00000000
FSL F4-0085	Paenibacillus odorifer	4	Pasteurized milk	6.8	80	SRR4242611	MPTE00000000
FSL F4-0087	*Paenibacillus* sp.	5	Pasteurized milk	6.9	70	SRR4434614	MRTE00000000
FSL F4-0100	*Paenibacillus* sp.	8	Pasteurized milk	7.7	72	SRR4434613	MRTF00000000
FSL F4-0126	Paenibacillus odorifer	13	Pasteurized milk	6.9	71	SRR4242620	MPTU00000000
FSL F4-0134	Paenibacillus odorifer	16	Pasteurized milk	6.9	72	SRR4242605	MPTJ00000000
FSL F4-0152	Paenibacillus odorifer	19	Pasteurized milk	7	105	SRR4242594	MKQK00000000
FSL F4-0242	Paenibacillus odorifer	25	Pasteurized milk	7.5	65	SRR4242600	MPTN00000000
FSL F4-0260	*Paenibacillus* sp.	29	Pasteurized milk	7	74	SRR4434612	MRTG00000000
FSL H3-0280	Paenibacillus odorifer	27	Raw milk	7.1	77	SRR4242617	MKQO00000000
FSL H3-0287	Paenibacillus odorifer	2	Raw milk	7	73	SRR4242603	MPTK00000000
FSL H3-0305	Paenibacillus odorifer	38	Pasteurized milk	7	153	SRR4434611	MPVM00000000
FSL H3-0464	Paenibacillus odorifer	46	Pasteurized milk	7.2	101	SRR4242597	MPTQ00000000
FSL H3-0465	Paenibacillus odorifer	50	Pasteurized milk	7	96	SRR4242622	MPTS00000000
FSL H7-0326	*Paenibacillus* sp.	57	Pasteurized milk	6	129	SRR4434610	MPVN00000000
FSL H7-0331	*Paenibacillus* sp.	58	Pasteurized milk	9.5	69	SRR4434619	MRTH00000000
FSL H7-0433	Paenibacillus odorifer	36	Pasteurized milk	7.5	54	SRR4242604	MPVP00000000
FSL H7-0443	*Paenibacillus* sp.	30	Pasteurized milk	6.9	58	SRR4242613	MPTM00000000
FSL H7-0604	Paenibacillus odorifer	74	Pasteurized milk	7.3	83	SRR4242618	MKQP00000000
FSL H7-0692	*Paenibacillus* sp.	80	Pasteurized milk	7	86	SRR4434623	MRTI00000000
FSL H7-0694	Paenibacillus odorifer	35	Pasteurized milk	6.8	75	SRR4242602	MPTL00000000
FSL H7-0710	*Paenibacillus* sp.	81	Pasteurized milk	6.7	104	SRR4242614	MPTC00000000
FSL H7-0713	Paenibacillus odorifer	33	Pasteurized milk	6.8	75	SRR4242601	MPTM00000000
FSL H7-0718	Paenibacillus odorifer	32	Pasteurized milk	7	77	SRR4242598	MPTP00000000
FSL H7-0744	*Paenibacillus* sp.	41	Raw milk	7.7	55	SRR4242615	MPTB00000000
FSL H7-0918	Paenibacillus odorifer	88	Pasteurized milk	7.3	89	SRR4242599	MPTO00000000
FSL H8-0069	Paenibacillus odorifer	93	Pasteurized milk	6.9	102	SRR4242610	MPTF00000000
FSL H8-0147	Paenibacillus odorifer	40	Pasteurized milk	7	66	SRR4242608	MPTH00000000
FSL H8-0175	Paenibacillus odorifer	107	Raw milk	7.1	89	SRR4242607	MPTI00000000
FSL H8-0237	Paenibacillus odorifer	15	Pasteurized milk	7.3	103	SRR4242619	MPTV00000000
FSL H8-0246	*Paenibacillus* sp.	108	Pasteurized milk	6.7	121	SRR4434621	MRTJ00000000
FSL H8-0259	*Paenibacillus* sp.	109	Pasteurized milk	8.2	74	SRR4434620	MRTL00000000
FSL H8-0548	*Paenibacillus* sp.	156	Water from hose, milking parlor	7.2	90	SRR4434627	MRTK00000000
FSL H8-0551	*Paenibacillus* sp.	157	Raw milk	6.1	102	SRR4434626	MRTM00000000
FSL J3-0120	*Paenibacillus* sp.	340	Pasteurized milk	6	93	SRR4434625	MRTN00000000
FSL J3-0122	*Paenibacillus* sp.	23	Pasteurized milk	7	87	SRR4434624	MRTO00000000
FSL J3-0153	Paenibacillus odorifer	46	Pasteurized milk	6.8	77	SRR4242596	MPTR00000000
FSL J3-0155	Paenibacillus odorifer	35	Pasteurized milk	7.1	60	SRR4242609	MPTG00000000
FSL J3-0159	Paenibacillus odorifer	7	Pasteurized milk	7	83	SRR4242616	MKQN00000000
FSL R5-0378	*Paenibacillus* sp.	214	Pasteurized milk	7.8	81	SRR4434628	MRTP00000000
FSL R5-0490	*Paenibacillus* sp.	93	Pasteurized milk	4.7	145	SRR4434636	MRTQ00000000
FSL R5-0527	Paenibacillus macerans	238	Pasteurized milk	8.4	77	SRR4434637	MRTR00000000
FSL R5-0636	Paenibacillus odorifer	180	Pasteurized milk	7	76	SRR4242595	MKQL00000000
FSL R5-0765	*Paenibacillus* sp.	261	Pasteurized milk	6.9	94	SRR4434634	MRTS00000000
FSL R5-0808	Paenibacillus glucanolyticus	159	Pasteurized milk	6.4	40	NA	ASPT00000000
FSL R5-0817	Paenibacillus glucanolyticus	159	Pasteurized milk	7.1	77	SRR4434635	MRTT00000000
FSL R5-0883	Paenibacillus odorifer	27	Pasteurized milk	7.2	77	SRR4242606	MKQM00000000
FSL R5-0923	*Paenibacillus* sp.	167	Pasteurized milk	7.1	91	SRR4242612	MPTD00000000
FSL R5-0937	Paenibacillus odorifer	21	Pasteurized milk	7.4	82	SRR4242621	MPTT00000000
FSL R7-0131	*Paenibacillus* sp.	179	Pasteurized milk	6.1	93	SRR4434632	MRTU00000000
FSL R7-0269	*Paenibacillus* sp.	163	Pasteurized milk	7.5	40	NA	ASPS00000000
FSL R7-0273	*Paenibacillus* sp.	193	Pasteurized milk	7.2	57	SRR4434633	MRTY00000000
FSL R7-0277	*Paenibacillus* sp.	45	Pasteurized milk	7.6	40	NA	ASPX00000000
FSL R7-0321	*Paenibacillus* sp.	199	Pasteurized milk	6.1	86	SRR4434630	MRTV00000000
FSL R7-0333	*Paenibacillus* sp.	201	Pasteurized milk	7.7	35	SRR4434631	MRTW00000000
FSL R7-0337	*Paenibacillus* sp.	202	Pasteurized milk	7.6	83	SRR4434638	MRTX00000000

a NA, SRR accession data are not available for these isolates (reported previously).

**FIG 2:**
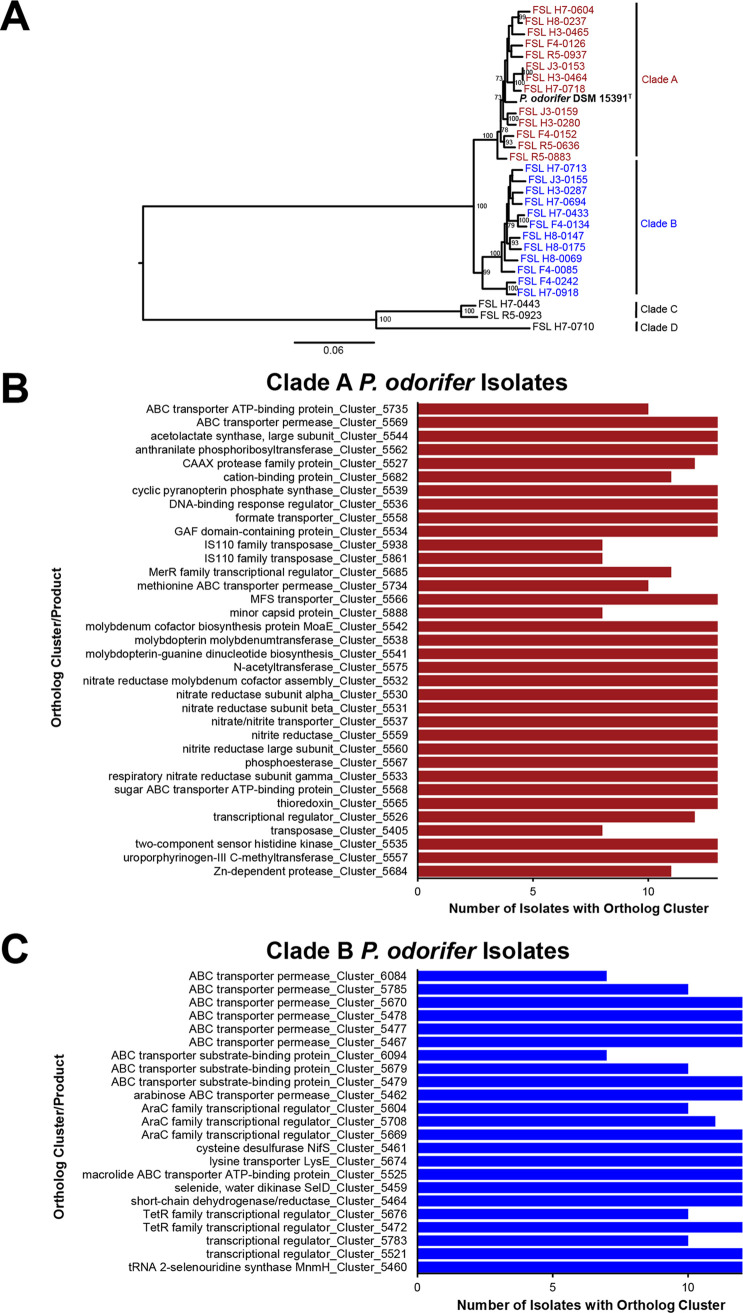
Whole-genome sequence comparison of 25 *P. odorifer* isolates. (A) Phylogenetic tree constructed from 19,990 core SNPs identified in the genomes of 25 *P. odorifer* isolates and 3 isolates representing closely related species (*Paenibacillus* sp. 5 [clade D] and sp. 6 [clade C]). The maximum likelihood tree was constructed using a general time-reversible model with gamma-distributed sites and 1,000 bootstrap repetitions. Only bootstrap values of ≥70 are shown. Isolates are colored by clade. (B and C) Bar charts showing the number of isolates that contain a given gene cluster/product that was not detected in any isolates in the other clade for isolates in clade A (B) and clade B (C). Ortholog clusters encoding hypothetical proteins that were uniquely present in each clade (*n* = 68 and 51 for clade A and B isolates, respectively) are not shown. Complete lists of all ortholog clusters and GO terms significantly enriched in clade A and B isolates are shown in Data Sets S1 through S4 in the supplemental material. MFS, major facilitator superfamily.

As *P. odorifer* represented the predominant *Paenibacillus* sp. isolated from milk, we performed further genetic analyses to characterize the diversity and genomic features of this species. WGS analyses of the 25 *P. odorifer* isolates and 3 isolates (representing isolates of *Paenibacillus* species 5 and 6) from closely related *Paenibacillus* species clades identified 19,990 core single-nucleotide polymorphisms (SNPs), resulting in four well-supported phylogenetic clades (Fig. 2A). Phylogenetic clade C, which contains isolates FSL H7-0443 and FSL R5-0923, represents unnamed *Paenibacillus* sp. 6 (Fig. 2A), while clade D, which contains a single isolate (FSL H7-0710), represents unnamed *Paenibacillus* sp. 5 (Fig. 2A). The remaining 25 isolates (representing 22 unique *rpoB* ATs) demonstrate the presence of two phylogenetic clades (clades A and B) of *P. odorifer.* Reexamination of the count data showed that clade A isolates accounted for 65% of all *P. odorifer* isolates (*n* = 536 isolates) in the data set, suggesting that among the *P. odorifer* isolates characterized here, clade A *P. odorifer* isolates are more commonly found in fluid milk and milk products than clade B isolates.

### A number of ortholog clusters and GO terms are significantly overrepresented in isolates within the two major *P. odorifer* clades.

Given the apparent genetic diversity observed among the *P. odorifer* isolates, we hypothesized that these two clades may have adapted to different ecological niches and that ortholog clusters or gene ontology (GO) terms associated with these clades may provide additional insight into the ecological niches that isolates in these clades might be associated with. The core genome for all 25 *P. odorifer* isolates characterized here included 4,562 genes, and the accessory genome (i.e., genes present in at least one *P. odorifer* genome) included 10,697 genes. Overall, 172 and 164 ortholog clusters and 94 and 52 GO terms were found to be significantly (false discovery rate [FDR]-corrected *P* value of <0.05) overrepresented among clade A and clade B isolates, respectively.

The 172 ortholog clusters overrepresented in clade A isolates included 116 clusters annotated as hypothetical proteins (see Data Set S1 in the supplemental material). In addition, six ortholog clusters annotated as encoding proteins with nitrate and nitrite reduction-related functions were found in all 13 clade A isolates but were absent from all 12 clade B isolates (Fig. 2B and Data Set S1). This is consistent with the observation that related GO terms {e.g., nitrate reductase [NAD(P)H], nitrogen metabolic processes, and the nitrate reductase complex} (Data Set S3) were also significantly overrepresented among clade A isolates. Similarly, three ortholog clusters were annotated as encoding MerR family transcriptional regulators, and an additional three clusters annotated as encoding proteins with molybdenum-associated functions were significantly overrepresented among clade A isolates (Fig. 2B and Data Set S1). Likewise, GO terms related to these processes (e.g., molybdopterin synthase activity and molybdopterin cofactor metabolic process) (Data Set S3) were also significantly overrepresented among clade A isolates, which is consistent with the presence of molybdopterin-containing nitrate reductases (4, 28, 29).

10.1128/mSphere.00739-19.3 DATA SET S1 Clade A enriched ortholog gene clusters in 25 *P. odorifer* isolates. Download Data Set S1, XLSX file, 0.02 MB. Copyright © 2020 Beno et al. 2020 Beno et al. This content is distributed under the terms of the Creative Commons Attribution 4.0 International license.

Clade B included 164 overrepresented ortholog clusters and 52 overrepresented GO terms. As with clade A isolates, many (*n* = 76) of the ortholog clusters overrepresented among clade B isolates represent hypothetical proteins (Data Set S2). In addition, six ortholog clusters of ABC transporter permeases and three ortholog clusters each of ABC transporter substrate-binding proteins and AraC family transcriptional regulators were overrepresented among clade B isolates (Fig. 2C and Data Set S3). GO terms related to these functions (e.g., UDP-glucose transport, UDP-glucose transmembrane transporter activity, and others) (Data Set S4) were also overrepresented among clade B isolates. Taken together, these analyses demonstrate previously undocumented intraspecies diversity among *P. odorifer* isolates commonly found in fluid milk.

10.1128/mSphere.00739-19.4 DATA SET S2 Clade B enriched ortholog gene clusters in 25 *P. odorifer* isolates. Download Data Set S2, XLSX file, 0.02 MB. Copyright © 2020 Beno et al. 2020 Beno et al. This content is distributed under the terms of the Creative Commons Attribution 4.0 International license.

10.1128/mSphere.00739-19.5 DATA SET S3 Clade A enriched GO terms in 25 *P. odorifer* isolates. Download Data Set S3, XLSX file, 0.02 MB. Copyright © 2020 Beno et al. 2020 Beno et al. This content is distributed under the terms of the Creative Commons Attribution 4.0 International license.

10.1128/mSphere.00739-19.6 DATA SET S4 Clade B enriched GO terms in 25 *P. odorifer* isolates. Download Data Set S4, XLSX file, 0.01 MB. Copyright © 2020 Beno et al. 2020 Beno et al. This content is distributed under the terms of the Creative Commons Attribution 4.0 International license.

### Genes associated with nitrogen fixation are highly conserved among *P. odorifer* isolates from fluid milk, but clade A isolates carry additional genes associated with nitrate and nitrite reduction.

**FIG 3:**
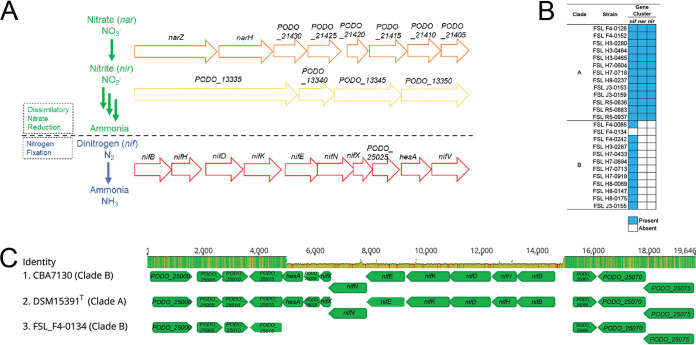
Clade A and B isolates encode different nitrogen metabolism-associated pathways. (A) Illustration of the gene clusters in *P. odorifer* strain DSM 15391^T^ associated with nitrate and nitrite reduction and nitrogen fixation; these genes were used for BLAST searches in panel B. (B) Presence and absence of the *nif* (nitrogen fixation), *nar* (nitrate reduction), and *nir* (nitrite reduction) gene clusters for all 25 *P. odorifer* isolates. (C) Alignment of 5′ and 3′ regions flanking the *nif* gene cluster in isolate FSL F4-0134, compared to closed genomes for DSM 15391^T^ (clade A; NCBI accession number NZ_CP009428.1) and CBA7130 (clade B; NCBI accession number GCA_003255855.1). Alignments were performed using Muscle in Geneious software for 10 iterations. Identity scores shown in green (top column) have 100% nucleotide sequence identity; regions shown in yellow and red have between 30 and 99% and ≤29% nucleotide sequence identities, respectively. Gene names represent those annotated in the closed genome for DSM 15391^T^.

As multiple nitrate- and nitrite-associated metabolic pathways were significantly enriched in clade A isolates (Fig. 2B and Data Sets S1 and S3), we also performed BLAST searches for additional nitrogen metabolic pathways (i.e., nitrous oxide and nitric oxide) (Fig. 3A and Table S2). Genes associated with nitrogen fixation were highly conserved (98 to 99% nucleotide identity with *nif* genes from *P. odorifer* DSM 15391^T^) in 24 *P. odorifer* isolates representing both clades A and B. For one clade B isolate (FSL F4-0134) (Fig. 3B), the *nif* gene cluster was not detected. Alignment of the 5′ and 3′ regions flanking the *nif* gene cluster in this isolate with 5′ and 3′ regions flanking the *nif* gene cluster in closed genomes for clade A (DSM 15391^T^) and clade B (CBA7130) strains confirmed the absence of *nif* genes in FSL F4-0134 (Fig. 3C). Searches for genes associated with nitrate and nitrite reduction (*nar* and *nir* gene clusters, respectively) (Fig. 3A) corroborated GO term enrichment analyses, as nitrate and nitrite reductase gene clusters were detected in all clade A isolates (Fig. 3B) but were not detected in any clade B isolates. These gene clusters were also highly conserved, having 99% nucleotide sequence similarity for both *nar* and *nir* gene clusters among all clade A isolates. Additional searches for genes associated with nitric oxide and nitrous oxide reduction were performed (Table S2), but these genes were not detected in any of the 25 *P. odorifer* genomes, regardless of the phylogenetic clade.

Given the dichotomy in the number of nitrogen metabolism gene clusters harbored among clade A and B isolates, we hypothesized that clade A isolates also differed phenotypically in their ability to utilize different nitrogen sources. Indeed, all 13 clade A isolates were able to reduce nitrate to nitrite within a 24-h incubation at 32°C, while none of the 12 clade B isolates were able to reduce nitrate to nitrite, even after approximately 96 h of incubation. *P. odorifer* isolates were also grown in the presence of nitrite, but none of the 25 isolates were able to reduce nitrite when grown at 32°C despite all clade A isolates containing genes with predicted nitrite reduction activity (Fig. 3B). Together, these results suggest that *P. odorifer* from milk shows clade-associated nitrate reduction activity.

### *P. odorifer* isolates encode multiple cold shock-associated genetic elements and have a conserved ability to grow at 6°C.

**FIG 4:**
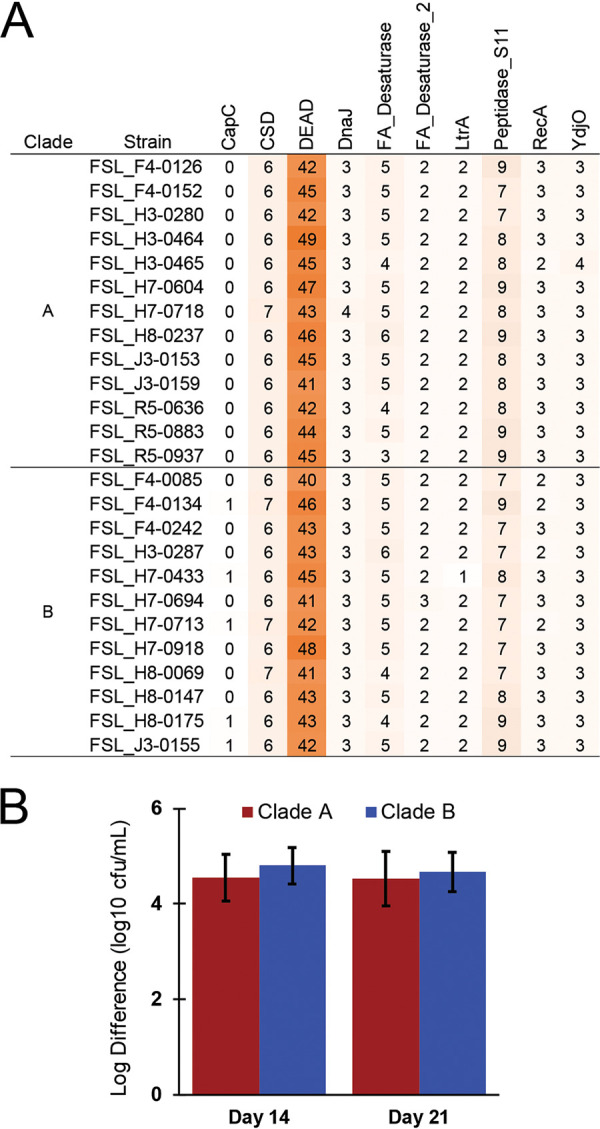
*P. odorifer* isolates encode a variety of cold shock-associated genetic elements and have a conserved ability to grow at 6°C in skim milk. (A) Heat map displaying the number of hits identified for cold shock-associated genetic elements (e.g., proteins, the cold shock domain, and DEAD box sequences) for each isolate. Values are shaded to reflect the relative number of sequences detected (orange indicates high, and white indicates low). (B) Log difference in CFU per milliliter for isolates grown in SMB at 6°C for 14 or 21 days, compared to the CFU per milliliter in the inoculum at day 0. Results are averaged for each clade and are shown for clade A (*n* = 13 isolates) and clade B (*n* = 12 isolates). Error bars represent the standard deviations of data from three independent replicates plated in technical duplicates for all isolates in the clade.

The ability to grow at refrigeration temperatures (6°C) requires certain physiological adaptations, such as alterations to the lipid composition of cellular membranes to retain fluidity (30), and specialized proteins (e.g., CSPs) devoted to overcoming challenges associated with DNA replication and gene expression at low temperatures (15). Given the frequency of *P. odorifer* isolation from fluid milk, we hypothesized that *P. odorifer* isolates are likely to carry genes associated with the cold shock response or certain adaptations permitting growth at refrigeration temperatures. Hidden Markov model (HMM) searches for proteins and protein domains associated with psychrotolerance identified hits in at least one genome for 10 of these HMMs (Fig. 4A), including (i) one HMM (CapC; Pfam identifier PF14102.5) that identified matches in 5 genomes (with one or two matches per genome) and (ii) nine HMMs that identified matches in all 25 genomes (i.e., DEAD box domain [PF00270.28], peptidase S11 [PF00768.19], cold shock domain [CSD] [PF00313.21], FA desaturase [PF00487.23], DnaJ [PF00226.30], YdjO [PF14169.5], FA desaturase 2 [PF03405.13], LtrA [PF06772.1], and RecA [PF00154.20]). In all cases, each HMM hit in a given genome represented a different gene, indicating that these searches identified multiple genes for a given cold shock-associated element rather than a single gene that contained multiple copies of a given domain. The HMM for the DEAD box protein motif (PF00270.28) identified between 40 and 49 matches per genome, while none of the other HMMs identified more than 10 hits in a given genome (Fig. 4A).

Given the conservation of multiple cold shock domain proteins, we further assessed the ability of the 25 *P. odorifer* isolates to grow in SMB at 6°C after 14- and 21-day incubations. All 25 *P. odorifer* isolates grew in SMB incubated at 6°C. The average log CFU per milliliter increases from the initial concentration in milk were 4.7 log CFU/ml (range, 3.7 to 5.8 log CFU/ml) and 4.6 log CFU/ml (range, 3.3 to 5.9 log CFU/ml) after 14 and 21 days of incubation at 6°C (Fig. 4B), respectively; the observed increase in log CFU per milliliter did not differ significantly between days 14 and 21 (*P* = 0.08). Furthermore, there were no clade-associated differences in the bacterial numbers reached after 14 days (*P* = 0.3157) or 21 days (*P* = 0.6871) of incubation for clade A versus clade B isolates. Taken together, these results suggest that the ability to grow at 6°C is both genetically and phenotypically conserved among *P. odorifer* isolates commonly isolated from fluid milk and milk products.

## DISCUSSION

Bacterium-induced spoilage of fluid milk is increasingly recognized as a major challenge for the dairy industry (10, 20). Furthermore, the growing demand for extended shelf life (ESL) milk (12) imparts an additional hurdle for milk quality by increasing the period of time postpasteurization that the milk must remain of sufficient quality for consumption. *Paenibacillus* species isolates are frequently isolated from fluid milk (10, 12, 31), yet few studies have examined the genetic and phenotypic diversity among these isolates. To this end, we characterized a preexisting collection of 1,228 *Paenibacillus* species isolates collected from milk and dairy environments and found that *P. odorifer* is the predominant *Paenibacillus* sp. isolated from raw and processed milk and milk products. We also identified a number of cold shock proteins that may be associated with the conserved ability of these isolates to grow at 6°C. Further genomic analyses identified additional nitrogen metabolic pathways that are present in clade A *P. odorifer* isolates, which corroborates their ability to reduce nitrate, an activity that is absent among all clade B isolates tested here. Overall, our data suggest that efforts aimed at reducing contamination with bacterial spores need to address contamination with *P. odorifer*, the predominant *Paenibacillus* sp. isolated from fluid milk in the United States.

### *Paenibacillus* spp. associated with fluid milk represent considerable species diversity that remains to be formally described.

While the genus *Paenibacillus* currently represents >240 species (3), including a number of species that have been previously associated with dairy spoilage (3, 10, 20), our study suggests that *Paenibacillus* isolates from fluid milk and dairy-associated environments represent considerable genomic diversity, especially considering that a number of these species have yet to be formally described. Specifically, based on ANIb analyses of the 58 *Paenibacillus* species isolates characterized here by WGS, these isolates represent 21 different species. Based on comparisons of type strains with available WGS data, only 3 of these 21 species could be identified as named species. While the 18 species for which a definitive species identification could not be assigned may include some species that have been previously described, but which do not have type strains with publicly available WGS data, it is likely that a number of these species represent new species of *Paenibacillus*. Interestingly, a study examining psychrotolerant spore-forming bacteria in ESL milk in Germany also found multiple potentially new *Paenibacillus* spp. (12), suggesting that milk may serve as an enrichment enabling the detection of novel *Paenibacillus* spp. Future work to clarify the taxonomy of the *Paenibacillus* isolates for these unnamed species will require WGS data for a number of type strains, which will not only enhance our understanding of *Paenibacillus* taxonomy and diversity but will also be essential to provide better tools to detect and prevent *Paenibacillus* contamination in fluid milk.

Despite this diversity, in our collection of 1,228 different *Paenibacillus* spp., we found that *P. odorifer* was the predominant species, more specifically, clade A *P. odorifer*, which was isolated nearly twice as often as clade B isolates among the collection of U.S. isolates analyzed here. Future studies analyzing the genetic diversity of *P. odorifer* isolated from milk from other geographic regions will be important for assessing whether *P. odorifer* clade A isolates are more common on a global scale. One study found that among raw milk samples collected from four different dairy plants in Germany, *P. amylolyticus*/P. xylanexedens was the predominant sporeformer isolated from raw milk (representing 48% of all isolates), but this species was never recovered from ESL pasteurized milk (12); however, *P. odorifer* was isolated from 33% of packaged ESL milks sampled in that study, providing further support for its importance as a fluid milk contaminant (12). Although *Paenibacillus* spore populations may differ by geographic region, multiple other studies have shown that *P. odorifer* is present in milk (raw and pasteurized) processed in Belgium (32); Switzerland, Austria, and Germany (33); and other regions in the United States (34), suggesting that controlling *P. odorifer* contamination of fluid milk is a global challenge.

### Genomic characterizations highlight the presence of two well-supported clades of *P. odorifer* and previously uncharacterized nitrogen metabolism genes.

Genomic analyses of core SNPs from 25 *P. odorifer* isolates identified two well-supported clades. Further comparative analyses showed that among the 336 ortholog clusters that were significantly overrepresented in either clade, 57% were annotated as hypothetical proteins. Among the ortholog clusters that could be assigned a specific function, our results highlighted the variable presence of nitrate and nitrite metabolism-associated genes as a prominent difference between clade A and B *P. odorifer* strains commonly found in fluid milk. Multiple *Paenibacillus* spp. (e.g., the species P. beijingensis [35], P. brasilensis [36], P. borealis [37], P. polymyxa [38], P. graminis [39], and others) were previously shown to convert atmospheric nitrogen into ammonia, potentiating their use as biofertilizers (3). Here, we show that genes encoding nitrogen fixation are highly conserved among *P. odorifer* isolates from both clades A and B (Fig. 3B). *P. odorifer* was initially described as having nitrate reductase activity and weak nitrogenase activity (i.e., nitrogen fixation activity) (39). Using a larger strain set, combined with both genetic and phenotypic assessments, our data suggest that the ability to reduce nitrate is restricted to clade A isolates. Indeed, the type strain (*P. odorifer* DSM 15391^T^) clusters with other clade A isolates (Fig. 2A), all of which were able to reduce nitrate to nitrite. Importantly, the results from these analyses provide further resolution of the phenotypic variation among *P. odorifer* isolates and suggest that nitrate reduction is variable in this species. Although nitrite reduction was not detected in any of the isolates tested here, it is possible that other environmental conditions (e.g., strictly anaerobic conditions, lower temperatures, or different growth media) are necessary to activate nitrite reduction among these isolates.

The observed association between the presence of nitrate and nitrite metabolism-associated gene clusters and the phylogenetic clade of the isolate may suggest that clade A and B *P. odorifer* isolates have adapted to two different environmental niches. *P. odorifer* was originally isolated from the roots of wheat plants in France (39) and has since been isolated from the rhizospheres of other plants in Brazil (40). While further research examining the environmental niches of *P. odorifer* will be beneficial for enhancing our understanding of any potential roles that *P. odorifer* plays in the nitrogen cycle, our results suggest that *P. odorifer* isolates may serve different functions in reducing nitrate and nitrite in the environment, reflected by the fact that clade A isolates carry genes for reducing nitrate and nitrite, while clade B isolates do not. While our gene presence/absence data suggest that clade A *P. odorifer* isolates may be better suited to anaerobic respiration than clade B isolates, further experiments assessing nitrite reduction in clade A strains grown under strictly anaerobic conditions are needed (41). While anaerobic growth was not quantified here, the *P. odorifer* type strain (clade A) has been previously reported to grow under anaerobic conditions in brain heart infusion (BHI) broth (39). As *Paenibacillus* spp. have been isolated previously from silage, a common feed for dairy cows, as well as from cow feces, it is possible that the additional nitrogen metabolic pathways encoded by clade A *P. odorifer* may facilitate its survival in these environments (42, 43). While further studies examining the potential roles that nitrate and nitrite reduction play in clade A *P. odorifer*’s adaptation to or survival under certain environmental conditions may be beneficial, reducing the transfer of *P. odorifer* from the environment into raw milk may require a multifaceted strategy to target both clade A and clade B isolates, as both can grow at 6°C in milk.

### *P. odorifer* isolates from raw and pasteurized milk show a uniform ability to grow at 6°C in skim milk, therefore controlling *P. odorifer* growth, and contamination is essential for reducing microbial outgrowth in fluid milk during shelf life.

Although *Paenibacillus* is one of the bacterial genera most commonly isolated from milk (10, 21), not all species/isolates are capable of growing in milk at refrigeration temperatures (18, 44). In this study, we found that all 25 *P. odorifer* isolates tested, representing 23 different *rpoB* allelic types, were capable of growing in SMB incubated at 6°C. Importantly, these data provide direct evidence that contamination with *P. odorifer*, regardless of the genotype of the isolate, is likely to result in outgrowth during refrigeration storage. Moreover, our data suggest that outgrowth of *P. odorifer* at refrigeration temperatures occurs within 14 days postpasteurization, as the average log increase after 14 days of incubation at 6°C was 4.7 log CFU/ml. A study by Buehler et al. showed that one *P. odorifer* isolate (FSL H3-0287, also included in this study) had a growth rate of 0.6 log CFU/ml per day in SMB incubated at 6°C, with an estimated lag-phase period of 1.9 days (44), suggesting that the isolates studied here likely increased by 4.7 log CFU/ml prior to our sampling at 14 days postinoculation. Previous characterizations of *P. odorifer* growth in fluid milk (10, 44) suggest that efforts to reduce the growth of bacterial spore-forming species in refrigerated fluid milk may be achieved by reducing the storage temperature. Reducing contamination with *P. odorifer* spores may be achieved at the farm level by implementing management practices aimed at reducing the transfer of bedding material, soil, and manure into raw milk, as these sources have been previously shown to harbor psychrotolerant spore-forming bacteria like *Paenibacillus* spp. (42, 45, 46). Finally, as *Paenibacillus* spp. are rarely isolated from ultrahigh temperature (UHT) milk, the use of higher pasteurization temperatures and/or the removal of spores using microfiltration (47) may be used as a processing control to reduce viable *Paenibacillus* species spores in fluid milk.

### CSPs are widely distributed in *P. odorifer* isolated from fluid milk, suggesting that multiple genetic elements are likely associated with psychrotolerance.

Here, we combined genetic screening for previously established cold shock-associated genetic elements with phenotypic growth studies to show that *P. odorifer* is well adapted to growth in milk at refrigeration temperatures. As *P*. *odorifer* spores survive traditional pasteurization temperatures (11), pasteurized milk provides an ideal medium for spore germination and outgrowth for *P. odorifer*. While we have previously shown that *Paenibacillus* spp. carry multiple cold shock-associated genetic elements (20), the analyses provided here demonstrate that *P. odorifer* isolates from fluid milk carry multiple copies of these elements. Among Bacillus cereus group isolates, which are also frequently isolated from pasteurized fluid milk and which may cause spoilage (48–50), a similar number of cold shock-associated genetic elements was found (51) compared to the *P. odorifer* isolates examined here. Importantly, among the B. cereus group isolates, only those from clade VI (representing Bacillus weihenstephanensis) were able to grow at 6°C (51) despite all isolates containing at least one copy of 12 different cold shock-associated proteins and genetic elements. Therefore, psychrotolerance among *P. odorifer* isolates, and other *Bacillales* such as the B. cereus group, is likely the sum result of multiple adaptations.

Overall, our data indicate that *P. odorifer* is the predominant *Paenibacillus* sp. isolated from fluid milk in the United States. Together, both the genetic conservation of multiple cold shock proteins and the phenotypic conservation of psychrotolerance demonstrated by all *P. odorifer* isolates characterized here suggest that, despite other genetic and phenotypic differences (i.e., nitrogen metabolism) between *P. odorifer* clades, *P. odorifer* is well suited to grow in refrigerated fluid milk.

## MATERIALS AND METHODS

### *Paenibacillus* species strain collection.

The 1,228 *Paenibacillus* species isolates described here were collated from previous (10, 11, 20, 21, 23, 24, 26), and ongoing (our unpublished work) studies. Isolates (see Data Set S5 in the supplemental material) were obtained from processed milk (defined here as in-process and pasteurized fluid milk and milk products) and raw milk as well as from dairy farm environmental samples (water, soil, teat end swabs, feed [corn silage, canola meal, flaked corn, and mixed-feed rations], and manure); all samples were collected in the United States. *Paenibacillus* species isolates that were sourced from raw milk were isolated from raw milk samples that were heat treated (80°C for 12 min or 100°C for 30 min) to select for spores. *rpoB* sequence data, including *rpoB*-based species and AT assignments, were also obtained from previous studies (10, 11, 20, 21, 23, 24, 26). Details on PCR amplification, sequencing, and analysis of *rpoB* sequence data for a 632-nucleotide (nt) internal fragment, used for species identification, have been previously described (10, 26, 52).

10.1128/mSphere.00739-19.7 DATA SET S5 Isolate information for 1,228 *Paenibacillus* species isolates included in the data set. Download Data Set S5, XLSX file, 0.1 MB. Copyright © 2020 Beno et al. 2020 Beno et al. This content is distributed under the terms of the Creative Commons Attribution 4.0 International license.

### DNA extraction and WGS of *Paenibacillus* spp.

Whole-genome sequence analysis was performed for a subset of 58 *Paenibacillus* species isolates. These 58 isolates (Table 1) represented 52 unique *rpoB* ATs, which were selected to include both commonly isolated ATs (33/50 ATs with ≥5 isolates) (Fig. 1A) and less-commonly isolated ATs (18/127 ATs with <5 isolates). Six ATs were represented by two isolates each (*n* = 6 strains, representing additional isolates of AT2, AT27, AT35, AT46, AT159, or AT179); these six ATs included 3/4 ATs with >50 isolates. DNA extraction was performed using the QIAamp DNA minikit (Qiagen, Valencia, CA) according to the manufacturer-provided protocol, which was modified to include an additional 45-min lysis step. Briefly, cultures of *Paenibacillus* species isolates grown overnight in brain heart infusion (BHI) broth (Becton, Dickinson, Franklin Lakes, NJ) were pelleted by centrifugation and subsequently lysed with 180 μl of lysozyme (20 mg/ml) by incubation for 45 min at 37°C. DNA was eluted twice in 50-μl volumes of Tris-HCl (pH 8.0). DNA purity was assessed using a Nanodrop spectrophotometer (Thermo Fisher Scientific, Waltham, MA), and the concentration of double-stranded DNA (dsDNA) was determined using a Qubit dsDNA high-sensitivity kit (Thermo Fisher Scientific). The concentration of dsDNA was adjusted to 1 ng/μl, and DNA samples were submitted to the Cornell University Institute of Biotechnology Genomics Facility (Ithaca, NY) for Nextera XT DNA library preparation. Samples were pooled and sequenced in two Illumina HiSeq 2500 rapid runs with 2- by 100-bp paired-end reads targeting 83× and 91× coverages, respectively.

### Read processing, quality control, genome assembly, and annotation.

Nextera XT adapters, as well as low-quality bases and reads, were trimmed using default settings of Trimmomatic v0.33 (53). The quality of the short reads was assessed using FastQC (v0.11.2) (Babraham Bioinformatics). Genomes were *de novo* assembled using SPAdes v3.6.2 with a variety of k-mer sizes (i.e., 21, 33, 55, 77, and 99) (54). QUAST was used to verify the quality of the assembled draft genomes (55). Average coverage was determined by mapping the reads against draft genomes using BBMap v35.49 and computing the average depth using SAMtools (56). Sequence reads were submitted to SRA (BioProject accession number PRJNA343020), and assembled draft genomes were submitted to the WGS NCBI database through the prokaryotic genome annotation pipeline (57).

### SNP detection, phylogeny construction, and average nucleotide identity by BLAST analyses.

Core SNPs were identified using kSNP3 with a k-mer size of 19, which was selected from the output of Kchooser (58). A maximum likelihood (ML) tree using core SNPs from the kSNP3 output was constructed in RAxML v8.0 (59) with a general time-reversible (GTR) model with gamma-distributed sites (GAMMA). The tree was rooted by midpoint, and bootstrap values were calculated based on 1,000 bootstrap repetitions. The phylogenetic tree was edited in FigTree v1.4.2. Average nucleotide identity by BLAST (ANIb) analyses were performed using ani.py to delineate species boundaries of all 58 *Paenibacillus* genomes, with default parameters, as described previously (60).

### Identification of proteins related to psychrotolerance.

Ten hidden Markov models (HMMs) (Table S1) were used to search for protein domains or proteins that were previously shown to be associated with psychrotolerance. HMMs were obtained from the Pfam 26.0 protein family database (61), and searches were performed using HMMER3 (62).

10.1128/mSphere.00739-19.1 TABLE S1 Accession numbers for hidden Markov models used to search for cold shock-associated elements. Download Table S1, DOCX file, 0.01 MB. Copyright © 2020 Beno et al. 2020 Beno et al. This content is distributed under the terms of the Creative Commons Attribution 4.0 International license.

### OrthoMCL and gene ontology term annotation.

OrthoMCL (63) was used with three inflation values (5, 3, and 1.5) to find ortholog clusters (groups of orthologous genes found across multiple isolates) in the 25 *P. odorifer* genomes. First, an inflation value of 5 was used to search for ortholog clusters among all 25 genomes. Additional searches with inflation values of 3 and 1.5 were used to identify ortholog clusters that were not identified when an inflation value of 5 was used. This approach was used to minimize the inclusion of more than one gene from the same genome in a given ortholog cluster. Finally, ortholog clusters with <25 genomes with an inflation value of 1.5 were identified, resulting in a total of 10,070 clusters.

One representative protein sequence from each cluster was selected for gene ontology (GO) annotation using Blast2GO (64). Protein sequences were first searched against the Swiss-Prot database (65). Genes that were not assigned a GO term were then searched against the RefSeq database (66). The outputs were combined, and the assigned GO terms were linked to their respective ortholog clusters and to each member of the ortholog cluster.

### Determination of core and pangenome sizes, gene presence/absence analysis, and gene enrichment.

Core genomes and pangenomes for the 25 *P. odorifer* isolates were defined using Roary (67) with default settings. Counts of genomes with (presence) or without (absence) a given ortholog cluster were summed for all clade A and clade B *P. odorifer* isolates; clades A and B were defined based on the ML phylogenetic tree. The presence/absence of GO terms was also tallied. Next, 2-by-2 contingency tables were generated for each GO term/ortholog gene cluster, and two-sided Fisher’s exact tests were performed to assess whether a GO term/ortholog cluster was significantly enriched among clade A or B isolates. Odds ratios were also computed, and *P* values were adjusted using the false discovery rate approach.

### BLAST detection of nitrate and nitrite gene clusters.

Nitrite and nitrate gene clusters (Table S2) were queried against the draft genomes for the 25 *P. odorifer* isolates using BLASTn version 2.3.0 with default parameters.

10.1128/mSphere.00739-19.2 TABLE S2 Nitrate loci used for BLAST detection of nitrogen metabolic gene clusters for 25 *P. odorifer* isolates. Download Table S2, DOCX file, 0.01 MB. Copyright © 2020 Beno et al. 2020 Beno et al. This content is distributed under the terms of the Creative Commons Attribution 4.0 International license.

### Quantification of growth at 6°C in SMB.

The 25 *P. odorifer* isolates characterized by WGS were also phenotypically tested for growth in SMB over a 21-day incubation period at 6°C, representing a slight temperature abuse in the United States. Briefly, isolates were streaked onto BHI agar plates from frozen glycerol stocks, and plates were subsequently incubated at 32°C for 20 to 24 h. For each of three cold growth replicates, a separate colony was streaked onto a new BHI agar plate, which was subsequently incubated at 32°C for an additional 20 to 24 h. Suspensions of each isolate were prepared using a sterile loop to inoculate 5 ml of phosphate-buffered saline (PBS) with confluent growth from BHI agar plates. Suspensions were adjusted to an optical density at 600 nm (OD_600_) of ∼0.1, representing approximately 1 × 10^7^ CFU/ml, and then serially diluted to reach an inoculum of approximately 1 × 10^5^ CFU/ml. A 100-μl aliquot of this suspension was added to prechilled (4°C) 10-ml aliquots of SMB. SMB was chosen as a model system in our study as it provides a standardized medium that mimics the composition of skim milk. SMB was previously used to assess growth and other characteristics of spoilage organisms relevant to fluid milk (10, 18, 20, 23, 68). Inoculation concentrations were confirmed by spiral plating of samples in technical duplicate onto BHI agar using the 50-μl exponential setting (Autoplate 5000; Advanced Instruments, Inc., Norwood, MA). Colonies were enumerated after 48 h of incubation at 32°C, using the Q-Count colony counter (Advanced Instruments, Inc.). Inoculated SMB tubes were incubated at 6°C (without shaking), and subaliquots were serially diluted and spread plated (100 μl) or spiral plated (50 μl), as described above, in technical duplicate onto BHI agar plates at both 14 and 21 days postinoculation. Colonies were enumerated following a 48-h incubation on BHI agar at 32°C using the Q-Count colony counter.

### Nitrate and nitrite reduction.

Phenotypic assessment of nitrate and nitrite reduction was performed for the 25 *P. odorifer* isolates using standard methods (69). Briefly, isolates were streaked from a frozen glycerol stock onto BHI agar plates, followed by incubation at 32°C for 18 to 24 h. Individual colonies (two tested per isolate) were patched onto BHI agar plates, which were incubated for an additional 20 to 24 h at 32°C. A 5-μl loopful of patched colonies was then inoculated into 5 ml of nitrate broth and 5 ml of nitrite broth (3 g/liter beef extract [VWR, Solon, OH], 3 g/liter enzymatic digest of protein [BD], and 1 g/liter either potassium nitrate [Alfa Aesar, Ward Hill, MA] or potassium nitrite [Ward’s Science, Rochester, NY]), followed by incubation at 32°C for 20 to 24 h under static conditions. Nitrate reagents A and B (Sigma-Aldrich) were added to 1-ml aliquots of cultures grown in nitrate and nitrite broth, which were observed for the presence of nitrite (red). Cultures that were unable to reduce nitrate to nitrite (i.e., turned red only after the addition of zinc dust) were incubated for an additional 70 to 72 h at 32°C and retested as described above. Nitrate and nitrite reduction analyses were performed in biological duplicate.

### Statistical analyses for growth in SMB.

All statistical analyses were performed using R statistical software. A linear regression model (lme4 package [70]) was used to assess statistically significant differences (at α = 0.05) between the log CFU per milliliter obtained on day 0 and the log CFU per milliliter obtained after 14 and 21 days of incubation at 6°C; “phylogenetic clade,” and “strain” and “replicate” were included as fixed and random effects, respectively.

### Data availability.

All sequencing data associated with the manuscript have been deposited in the NCBI database (see Table 1 for accession numbers); all raw data are available upon request.
