# Supplementary material for: Paenibacillus odorifer, the Predominant Paenibacillus Species Isolated from Milk in the United States, Demonstrates Genetic and Phenotypic Conservation of Psychrotolerance but Clade-Associated Differences in Nitrogen Metabolic Pathways
Source: mSphere. 2020 Jan 22;5(1):e00739-19. doi: 10.1128/mSphere.00739-19 (PMC7407005; doi:10.1128/mSphere.00739-19)
Supplement: TABLE S1 [file mSphere.00739-19-st001.docx]

Supplementary Table S1. Accession numbers for Hidden Markov Models used to search for cold shock associated elements.

| Gene Cluster Product | Accession Number |
| --- | --- |
| Caps_synth_CapC | PF14102.5 |
| CSD | PF00313.21 |
| DEAD | PF00270.28 |
| DnaJ | PF00226.30 |
| FA_desaturase | PF00487.23 |
| FA_desaturase_2 | PF03405.13 |
| LtrA | PF06772.10 |
| Peptidase_S11 | PF00768.19 |
| RecA | PF00154.20 |
| YdjO | PF14169.5 |
