# Supplementary material for: Paenibacillus odorifer, the Predominant Paenibacillus Species Isolated from Milk in the United States, Demonstrates Genetic and Phenotypic Conservation of Psychrotolerance but Clade-Associated Differences in Nitrogen Metabolic Pathways
Source: mSphere. 2020 Jan 22;5(1):e00739-19. doi: 10.1128/mSphere.00739-19 (PMC7407005; doi:10.1128/mSphere.00739-19)
Supplement: TABLE S2 [file mSphere.00739-19-st002.docx]

Table S2. Nitrate loci used for BLAST detection of nitrogen metabolic gene clusters for 25 *P. odorifer* isolates.

| Gene Cluster Product | Nucleotide Positions | Strain | NCBI Accession |
| --- | --- | --- | --- |
| *nir* (nitrite reduction) | 3,012,807 – 3,017,234 | *Paenibacillus odorifer* DSM 15391^T^ | CP009428.1 |
| *nar* (nitrate reduction) | 4,761,372 – 4,770,630 | *Paenibacillus odorifer* DSM 15391^T^ | CP009428.1 |
| *nif* (nitrogen fixation) | 5,566,562 – 5,577,495 | *Paenibacillus odorifer* DSM 15391^T^ | CP009428.1 |
| *nor* (nitric oxide reduction) | 2,040,162 – 2,043,624 | *Bacillus licheniformis* ATCC 14850 | CP034569.1 |
| *nos* (nitrous oxide reduction) | 1,829,858 – 1,835,572 | *Geobacillus thermodenitrificans* NG80-2 | CP000557.1 |
